# Possible Further Evidence of Low Genetic Diversity in the El Sidrón (Asturias, Spain) Neandertal Group: Congenital Clefts of the Atlas

**DOI:** 10.1371/journal.pone.0136550

**Published:** 2015-09-29

**Authors:** Luis Ríos, Antonio Rosas, Almudena Estalrrich, Antonio García-Tabernero, Markus Bastir, Rosa Huguet, Francisco Pastor, Juan Alberto Sanchís-Gimeno, Marco de la Rasilla

**Affiliations:** 1 Group of Paleoanthropology, Department of Paleobiology, Museo Nacional de Ciencias Naturales, Madrid, Spain; 2 Department of Physical Anthropology, Aranzadi Society of Sciences, Donostia, Basque Country, Spain; 3 Institut Catalá de Palecologia Humana i Evolució Social IPHES, Area de Prehistoria, Universidad Rovira i Virgili, Unidad Asociada al CSIC, Tarragona, Spain; 4 Museo Anatómico, Department of Anatomy and Radiology, Faculty of Medicine, Universidad de Valladolid, Valladolid, Spain; 5 Department of Anatomy and Human Embriology, Faculty of Medicine, Universidad de Valencia, Valencia, Spain; 6 Área de Prehistoria, Department of History, Universidad de Oviedo, Oviedo, Spain; University of Kansas, UNITED STATES

## Abstract

We present here the first cases in Neandertals of congenital clefts of the arch of the atlas. Two atlases from El Sidrón, northern Spain, present respectively a defect of the posterior (frequency in extant modern human populations ranging from 0.73% to 3.84%), and anterior (frequency in extant modern human populations ranging from 0.087% to 0.1%) arch, a condition in most cases not associated with any clinical manifestation. The fact that two out of three observable atlases present a low frequency congenital condition, together with previously reported evidence of retained deciduous mandibular canine in two out of ten dentitions from El Sidrón, supports the previous observation based on genetic evidence that these Neandertals constituted a group with close genetic relations. Some have proposed for humans and other species that the presence of skeletal congenital conditions, although without clinical significance, could be used as a signal of endogamy or inbreeding. In the present case this interpretation would fit the general scenario of high incidence of rare conditions among Pleistocene humans and the specific scenariothat emerges from Neandertal paleogenetics, which points to long-term small and decreasing population size with reduced and isolated groups. Adverse environmental factors affecting early pregnancies would constitute an alternative, non-exclusive, explanation for a high incidence of congenital conditions. Further support or rejection of these interpretations will come from new genetic and skeletal evidence from Neandertal remains.

## Introduction

A better understanding of the process of origin, change and extinction of *Homo neanderthalensis*is accomplished through the study of its population dynamics. This objective can now be addressed through paleodemographic studies based on osteological data from the fossil record [1], through analysis of Neandertal genetic data [2, 3] and through a combination of statistical methods considering both kinds of information [4, 5].Besides this approach, based on gathering available data from chronologically and geographically dispersed fossils, it is also informative to investigate the structure and dynamics of groups composed of contemporaneous individuals, which represent a more fundamental unit of genetic and social organization of Neandertal populations. The peculiarities of the El Sidrón site (Asturias, Northern Spain), allow us to address these questions.

The El Sidrón Neandertal assemblage is composed of more than 2400 human fossils recovered ina secondary context in a karst system [6, 7], and dated to 49000 years ago[8]. A minimum number of thirteen individuals have been identified in this sample, including seven adults, three adolescents, two juveniles and one infant, with representation of all skeletal regions[9, 10].The genetic analysis of the sample indicated the presence of a small patrilocal community with low genetic diversity [11], corroborating previous archaeological and geological data that pointed to a contemporaneous social Neandertal group. Thus, besides genetic studies associated with diverse functional traits in Neandertals (i.e. pigmentation, blood group, language and taste perception) [12], the presence of this assemblage has allowed us to address important questions at the intraspecific and intra-group levels. The analysis of dental calculus has revealed evidence of cooking and plant use[13], further studies of the dentition have addressed sexual division of labor [14] and handedness[15], and morphological variability has been studied in different bone elements at the cranial [16, 17] and postcranial[18, 19]levels.

The presence of a contemporaneous Neandertal group with close genetic relations, together with recent studies in Neandertalpaleogenetics pointing to long-term small and decreasing population size with reduced and isolated groups [20], opens the possibility to explore the presence of skeletal traits related to kinship [21], and/or considered as potential signals of inbreeding[22, 23], supplementing genetic research in Neandertals regarding their biological variability and demography. In this regard, evidence of a retained deciduous mandibular canine, a condition with a probable familial basis, was reported for two dentitions from El Sidrón[24]. We present here the study of congenital clefts in the first cervical vertebrae within this groupand discuss its potential implications for Neandertal demography.

## Material and Methods

### Neandertal sample

**Fig 1:**
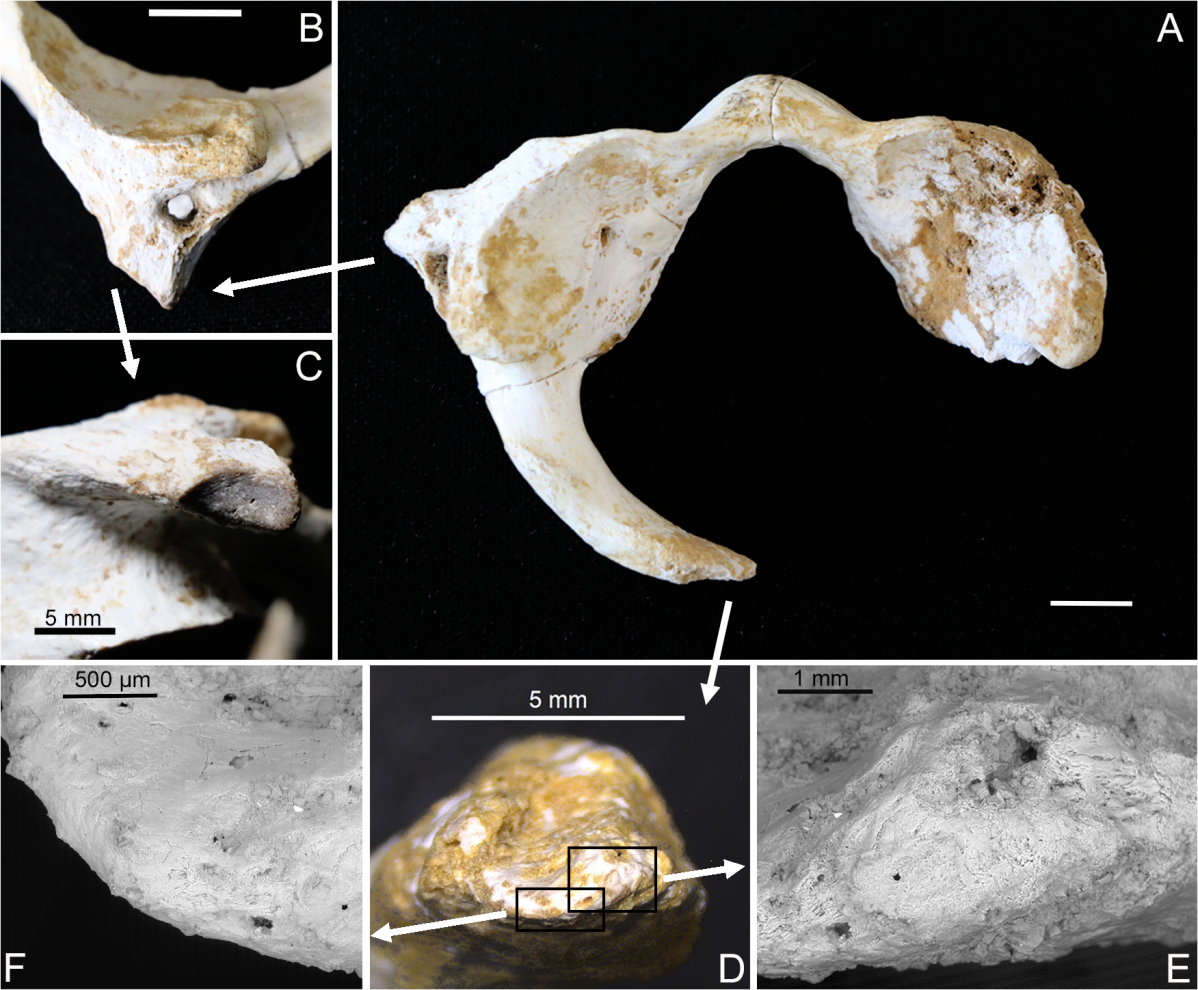
Atlas SD-1643. (A) Superior view of SD-1643, bar represents 1 cm. (B) Left transverse foramen completely closed, bar represents 1 cm. (C) Visible epiphyseal surface on the tip of the left transverse process. (D) Medial view of the tip of the left lamina, where no bone breakage and a smooth continuity of the cortical bone can be observed. (E) ESEM image, detail of the tip, with continuous cortical bone. (F) ESEM image, detail of another area of the tip with continuity of the cortical bone along its edge.

SD-1643 is an almost complete atlas reconstructed from three bony elements (Fig 1). It only lacks the right lamina or right posterior arch of the atlas due to a post-mortem fracture (transversal, regular surface of breakage with exposure of trabeculae) observed immediately posterior to the right lateral mass. The left lamina is truncated at the sagittal midline, the location of the posterior synchondrosis of the atlas (hereafter PS).

**Fig 2:**
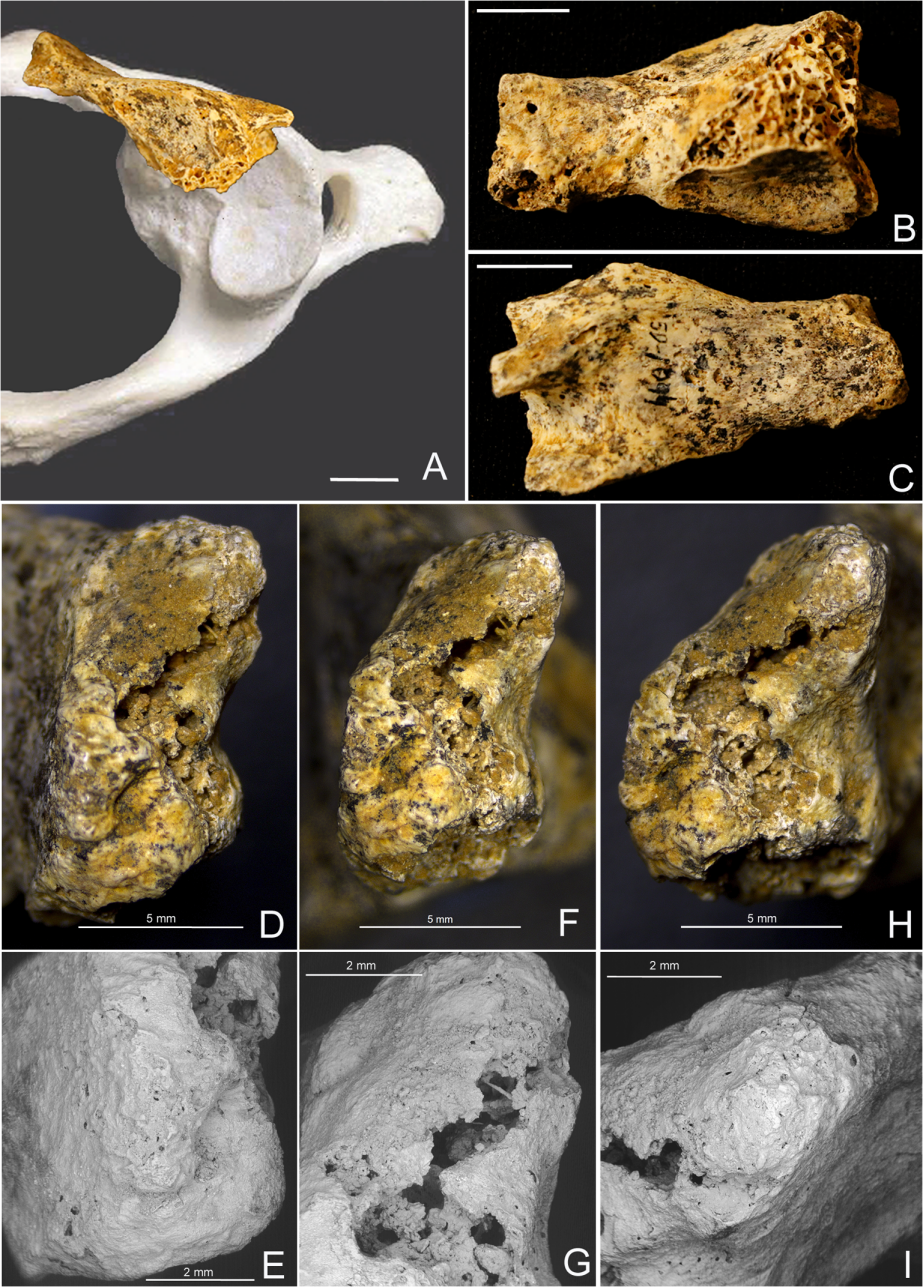
Atlas SD-1094. **(**A) Superior view of SD-1094, placed on its anatomical location within a modern human atlas, bar represents 1 cm. (B) Posterior view of SD-1094, the right superior and inferior articular surfaces can be observed. The facet for the dens of the axis can be observed in the anterior arch, bar represents 1 cm. (C) Anterior view of SD-1094, the beginning of the anterior tubercle of the transverse foramen can be observed on the left. (D) Medial anterior view of the midsagittal cleft, slightly tilted to superior in order to view its anterior inferior corner. Continuity of the cortical bone from the anterior to the sagittal surface can be observed. (E) ESEM image, with clearer view of the anterior inferior corner, with continuity of the cortical bone from the anterior to the sagittal surfaces. (F) Medial view of the anterior sagittal cleft. Cortical bone can be observed in the inferior and superior thirds. Bone breakage can be observed in the central third. (G) ESEM image, with clearer view of the cortical bone and the areas of bone breakage. (H) Medial posterior view with continuity of cortical bone from the posterior to the anterior surfaces of the posterior arch of the atlas. (I) ESEM image of the superior corner of the midsagittal cleft, with clearer view of the cortical continuity between the posterior and anterior surface along the sagittal cleft.

SD-1094 is a 33.1 mm length fragment whose location corresponds to the right anterolateral quadrant of a first cervical vertebra. The anterior half of the superior and inferior articular surfaces, as well as the facet for the dens of the axis, are recognizable (Fig 2). The anterior arch of the atlas truncates approximately at the sagittal midplane, whereas during development the anterior arch presents two synchondrosis (hereafter AS), each one located symmetrically medial to the lateral masses of the atlas.

**Fig 3:**
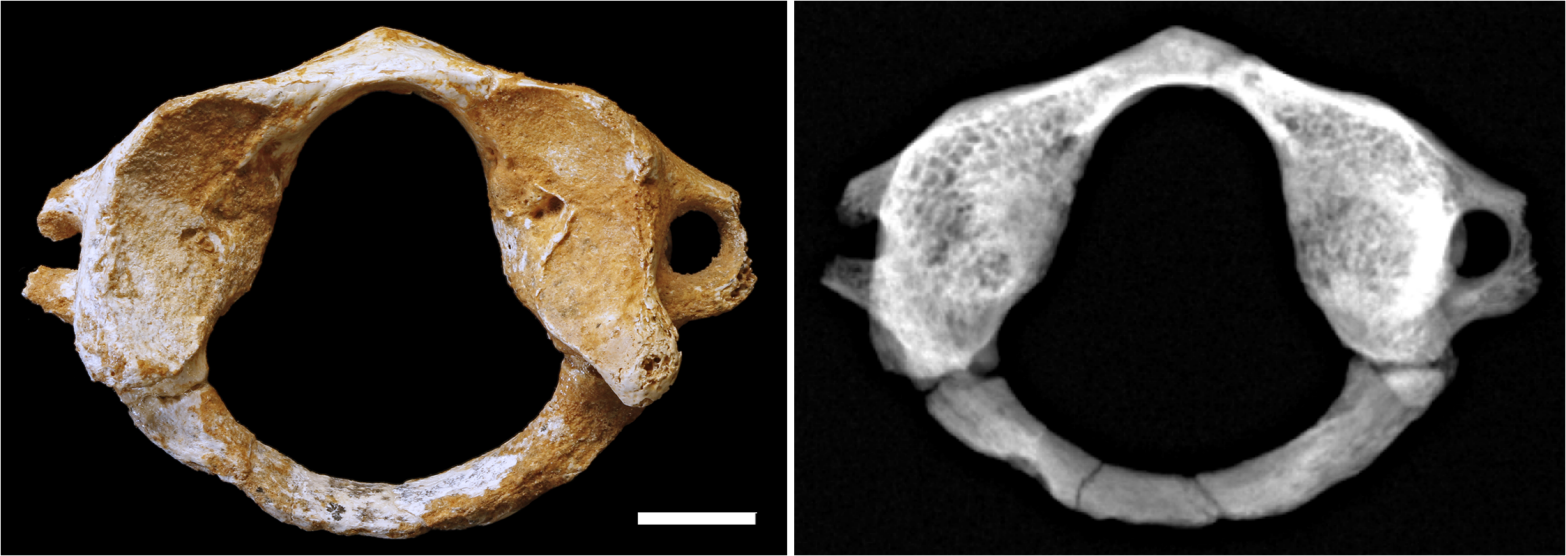
Atlas SD-1605/1595. (A) Superior view of SD-1605/1595, bar represents 1 cm. (B) Radiography of SD-1605/1595.

SD-1605/1595 is a complete adult atlas with no significant observable feature (Fig 3)

### Human sample

An extant modern human subadult sample comprised of documented and archaeological skeletons was gathered for comparative purposes. The documented skeletons come from the Museu Nacional de Historia Natural of Lisbon (Portugal), and from the Museo Anatómico from the Faculty of Medicine of Valladolid (Spain), with a total of 47 skeletons with an age range from birth to 14 years old. These known sex and age skeletonscomprised the skeletal documented sample (SDS). The 25 archaeological skeletons, ranging in dental age from 3.5 to 11.5 years[25], come from three different collections: Leiria (Portugal, 13^th^ to 16^th^Centuries) [26], Lagos (Portugal, 15^th^ to 17^th^ Centuries) [27],and Baza (Spain, 11^th^ to 16^th^ Centuries) [28]. The degree of closure of the AS and PS, and the transverse foramen (hereafter TF) of the atlas were recorded according to a three grade scale (0: absence of fusion; 1: active fusion; 2 complete fusion). Three adult human atlases with congenital clefts at the PS were collected for comparative purposes from the anatomical collection curated at the Anatomical Museum from the Faculty of Medicine, Valladolid (Spain).

### Methods

The regions of interest of SD-1643, SD-1094 and selected modern human atlases were inspected under binocular lens and Environmental Scanning Electron Microscope (ESEM Fei-Quanta 200) located at the National Museum of Natural History (MNCN-CSIC). Selected atlases were examined at 25.0 kv accelerating voltage and low vacuum mode. The magnification observations ranged from 40x to 1000x. Conventional radiographs were taken of the three Neandertal atlases and selected modern human atlases.

## Results and Discussion

The explanation of the truncation of the left lamina of SD-1643, and the anterior arch of SD-1094 is discussed via a differential diagnosis including normal-for-age lack of fusion of the PS and AS, ante-, peri- and post-mortem fractures, and congenital clefts of the arch of the atlas. Each of these possibilities is discussed separately.

### Normal-for-age lack of fusion of PS and AS

**Fig 4:**
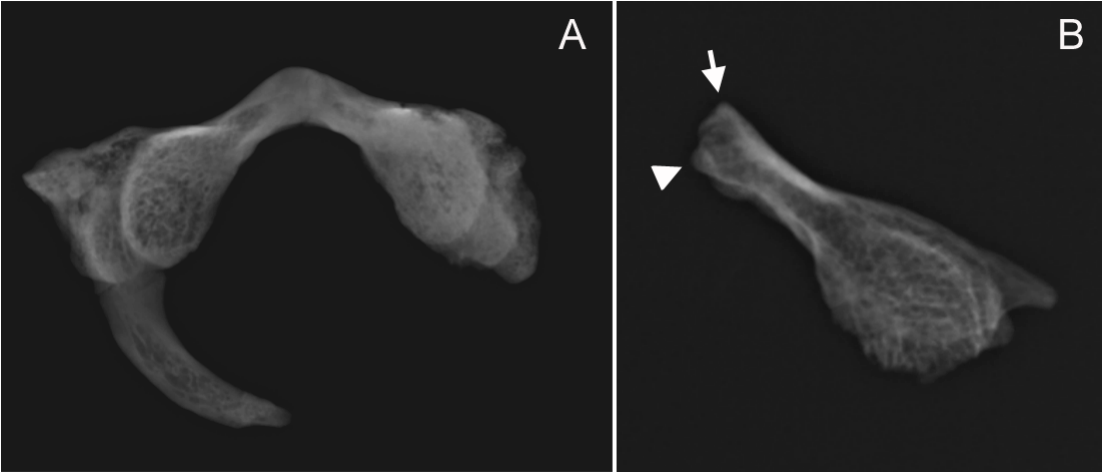
Radiographic images of SD-1643 and SD-1094. **(A)** Radiography of SD-1643, with continuity of the cortical bone through the irregularly-shaped tip of the left lamina. (**B)** Radiography of SD-1094, the arrow indicates the continuity of cortical bone at the anterior inferior corner of the midsagittal cleft (see Fig 2D and 2E), while the triangle indicates the continuity of cortical bone at the superior corner of the cleft (see Fig 2H and 2I). Lack of continuation of cortical bone through the entire cleft is due to post mortem breakage (see Fig 2F and 2G).

**Fig 5:**
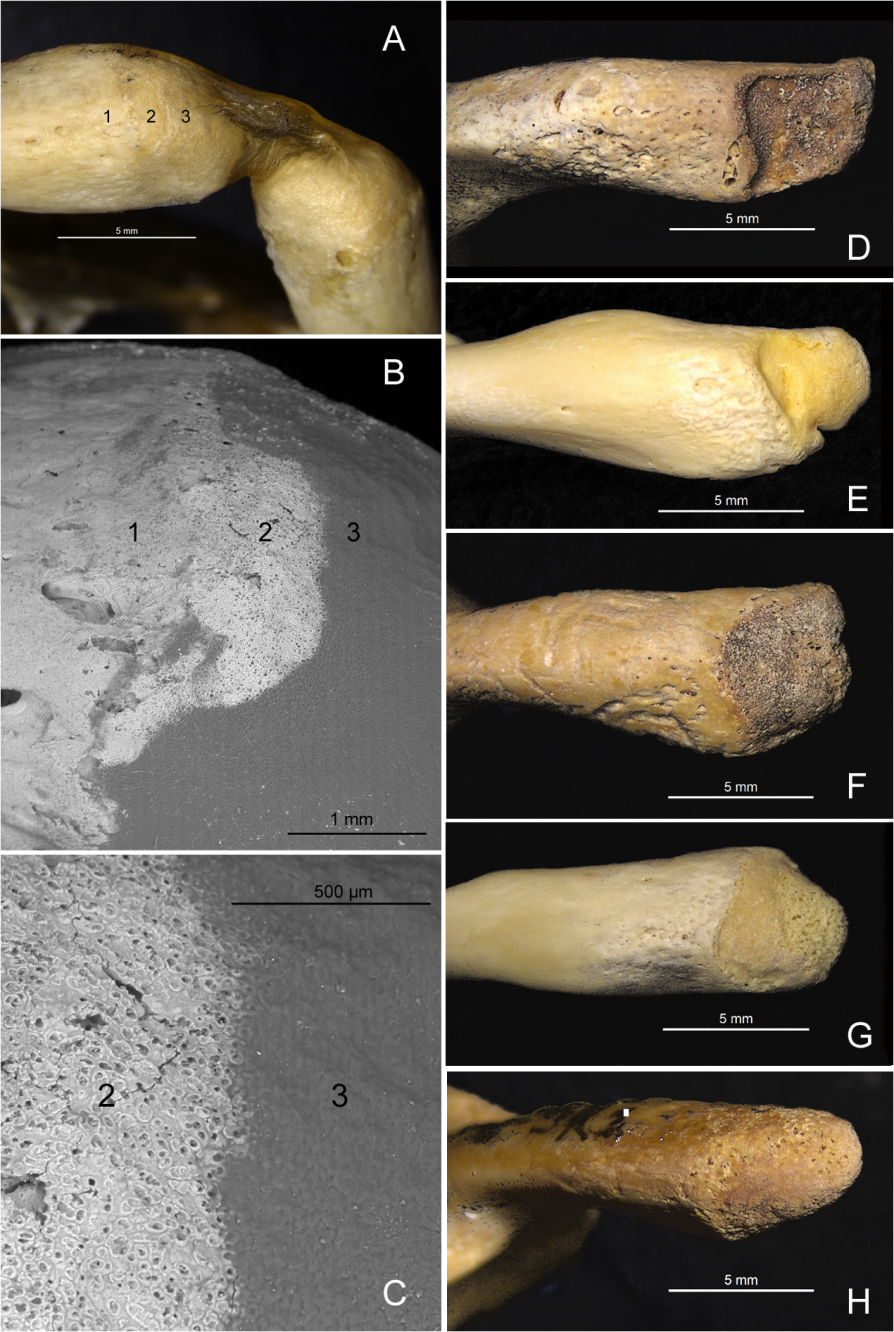
Non-fused posterior synchondrosis of the atlas. (A)The PS of the atlas of a 3 year old skeleton from the anatomical collection curated at University of Valladolid. Cartilage was left in place in this preparation and it can be observed bridging both tips of the posterior arches. Numbers are located in an area of the synchondrosis where a small portion of the cartilage has been lost. Numbers indicate: 1, cortical bone; 2, synchondrosis surface; 3, cartilage. (B) ESEM image with detail of the aforementioned area. Smooth surface of the cortical bone can be observed on the left (1). The surface of the synchondrosis presents a uniform porous aspect formed by numerous and small pore-like structures (2). The cartilage presents a uniform opaque surface (3). (C) ESEM image with further detail of the image presented in B. The porous-like surface of the synchondrosis (2) can be clearly observed, associated to the anchorage of the cartilage. (D, E, F, G, H)The PS (left posterior arch) of non-fused atlases from modern human subadult skeletons. The limit between the cortical bone and the synchondrosis surface is very diverse as can be observed in this small sample, but the differentiation between both surfaces is clear.

A normal-for-age lack of fusion of the PS for SD-1643 is excluded based on three observations. First, a universal feature of the pattern of maturation of the atlas in humans is the fact that the PS fuses invariably before the two AS [29–33], except in some atlases with only one sagittal AS, which is not the case of SD-1643. Second, the continuity of the cortical surface along the tip of the lamina can be observed in the radiograph(Fig 4). Third, absence of a synchondrosis-like surface on the tip of the lamina can be observed, indicating that there was no cartilage bridging the left lamina to the right lamina and thus there was no actual synchondrosis (Fig 5).Additional observations can be added regarding the maturation and chronological age of SD-1643 by modern human references, based on the degree of closure of the AS, the TF, and the degree of maturation of the epiphyseal surface located in the tip of the left transverse process (Fig 1). With regard to the AS, diverse CT scan studies indicate that the earliest age of closure is reported to be at 3–4 years [31, 33], while the oldest age at which the AS is apparent ranges from 6.83 years[33] to 7 years and 1 month [34] to 7 years and 3 months [31]. Our own observations in the SDS indicate that the oldest case of lack of fusion of the AS was 5.8 years, active fusion was observed between 5.3 to 7.2 years, and the earliest case of complete fusion was 6.1 years. With regard to the TF, it has been stated that it is “usually near completion by years 3–4” [35]. Our own observations in the SDS indicate that active formation of the TF was observed between 1.5 and 9 years, while the earliest case of complete TF was observed at 4.75 years. With regard to the epiphyseal surface of the tip of the transverse process, it has been observed that its presence indicates an age below 18 years, while the transformation of this surface into cortical bone indicates an age older than 15 years [36]. A broad age range from 4.75 years to 18 years can thus be assigned to SD-1643 based both on the complete closure of the TF and on the presence of the epiphyseal surface in the tip of the transverse process.

With regard to SD-1094, from a maturation perspective the frequency of mid-sagittal anterior clefts of the atlas in subadult samples ranges from 0.7% [31], to 16.5% [32], to even 21.8% [30], (observations limited to ages below 7 years [31] or below a maximum of 12 years [32]), indicating that during the subadult period the presence of only one AS at the anterior sagittal midline represents a normal variant of the maturation of the anterior arch of the atlas. Frequency values for adult samples range from 0.087% [37] to 0.1% [38], a fact that indicates that most of the anterior clefts observed in the subadult samples eventually ossify before adulthood [31]. Again, a normal-for-age lack of fusion of the AS for SD-1094 is excluded based on the continuity of the cortical bone and the absence of a synchondrosis-like surface on the midsagittal plane of the anterior arch, as observed in the ESEM and radiographic images (Figs 2, 4 and 5).

### Fractures of the atlas

For SD-1643, the possibility of an ante-, peri- or post-mortem fracture is discarded based on several observations. Different classifications of fractures of the atlas in the living have been developed based on their location [39–44], but no one includes a single fracture at the posterior sagittal midline. This fracture can occur, but in all the cases reviewed it is accompanied by at least one other fracture generally at the anterior sagittal midline [45], a combination included in some of the aforementioned classification systems [44]. Furthermore, as indicated above, radiography and observations through binocular lens and ESEM show two features: First, a clear smooth continuity of the cortex along the tip of the lamina of SD-1643, without exposure of the inner trabeculae, thus excluding peri- and post-mortem fractures; second, absence of osteogenic reaction thus excluding a healed but unfused ante-mortem fracture [46] (Fig 1).

With regard to SD-1094, fractures at the anterior sagittal midline have been described [45], but the augmented images again indicate a smooth continuity of the bony cortex from the ventral and dorsal surface of the anterior arch to the sagittal midplane (Fig 2). This continuity is only interrupted due to post-mortem erosion with exposed trabeculae in the central part of the midplane, and absence of osteogenic response compatible with a healed but unfused ante-mortem fracture is observed also for SD-1094 [46](Fig 2). These observations exclude ante-, peri- or post-mortem fractures as possible explanations for the presence of the sagittal midplane truncation of the anterior arch of the atlas of SD-1094.

### Congenital clefts of the atlas

**Fig 6:**
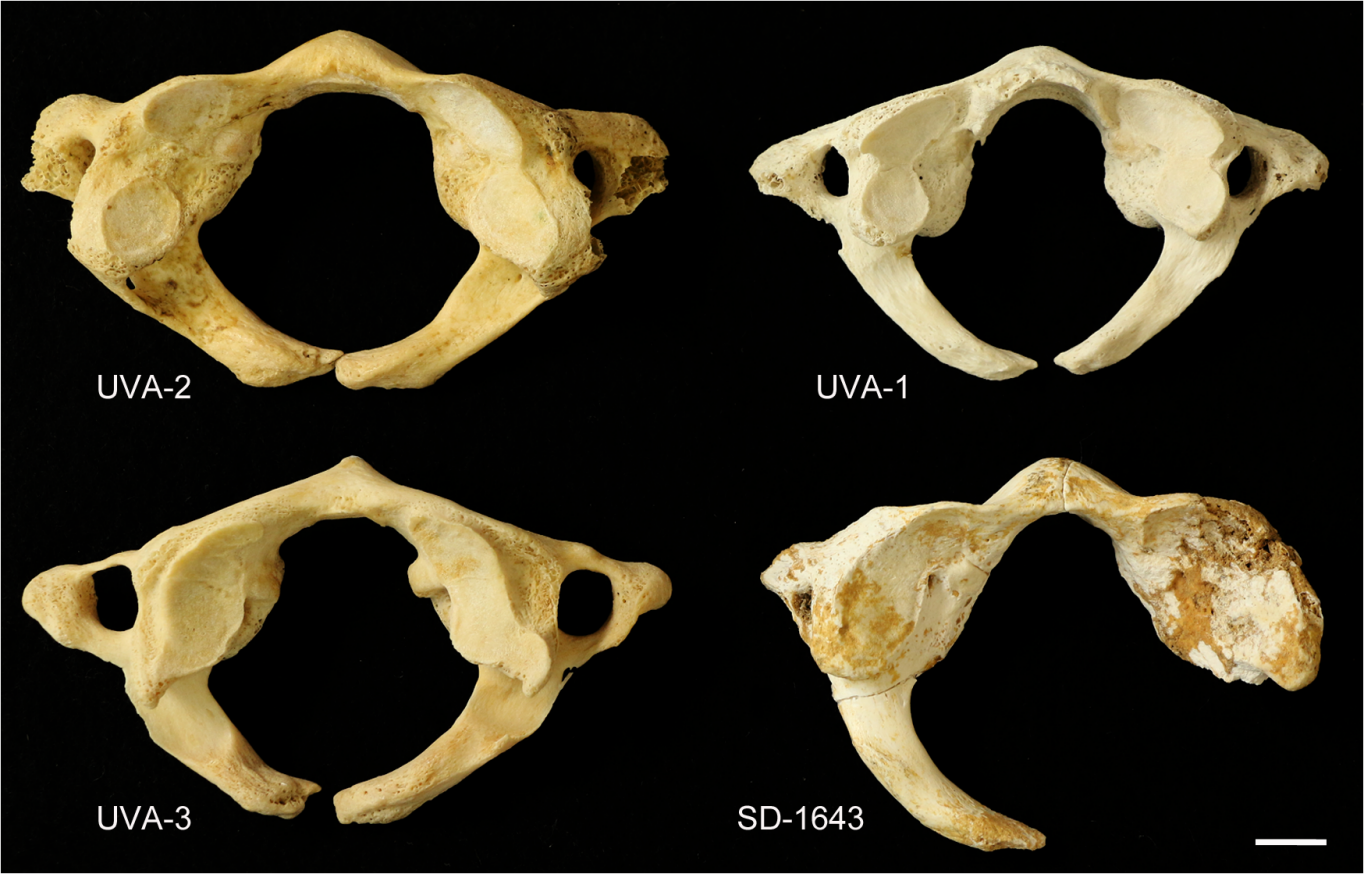
Atlases with type A defect. Comparison between SD-1643 and three cases of a type A posterior cleft of the atlas from the anatomical collection curated at University of Valladolid (UVA). In this superior view the truncation of the anterior arches at the midsagittal line can be observed. As indicated in the text, the post mortem breakage immediately posterior to the right lateral mass of SD-1643 opens the possibility of a type A or type B (absence of one lamina) defect.

The above findings indicate that the truncation of the left lamina of SD-1643 at the sagittal midline correspond to a congenital defect that could be classified as type A according to the system of Currarino et al. [47]. A comparison of SD-1643 with modern human cases with type A defects is presented in Fig 6. A type B defect is defined as a condition affecting only one lamina and ranging from a small gap to a complete absence of the lamina, and it cannot be excluded for SD-1643 due to postmortem absence of the right lamina (Fig 1). The frequency of type A defects ranges from 0.73% [48] to 3.84% [38], accounting for more than 77% of all of the defects reported for the posterior arch of the atlas [49–55] (Table 1).

**Table 1:** Frequency of congenital defects of the atlas in modern humans.

REFERENCE	SAMPLE	TYPE	N	TYPE-A	TYPE-A(%)	ALL DEFECTS (%)
[49]	USA	Clinical CT	839	26	3.1	-
[50]	Switzerland	Clinical CT	1069	34	3.18	3.8
[48]	Spain	Skeletal	136	1	0.73	0.73
[37]	Korea	Clinical CT	1153	9	0.78	0.95
[51]	USA	Clinical CT	1104	29	2.6	3.35
[51]	USA	Cadaveric	84	3	3.57	3.57
[47]	USA	Clinical (radiography & CT)	7200	-	-	0.069
[52]	French	Skeletal	500	14	2.8	2.8
[53]	European South African	Clinical radiographic	220	7	3.18	3.63
[54]	French	Skeletal	300	9	3	-
[38]	German	Cadaveric	1613	62	3.84	4
[55]	European	Skeletal	1626	26	1.59	-

With regard to SD-1094, the above findings indicate that the truncation of the anterior arch at the sagittal midplane corresponds to a congenital cleft, in this case a very rare condition in adults according to previous work, with reported frequencies from 0.087% [37], to 0.09% [50] to 0.1% [38]. It is also interesting to note that in most cases, an anterior cleft is associated with the presence of a posterior cleft resulting in a bipartite atlas [56–72]. With regard to clinical manifestations of defects of the arch of the atlas, they range from lack of symptoms even in cases of total aplasia of the posterior arch [73] to atlantoaxial instability [74] and cervical myelopathy (types C and D)[75]. Defects of the arch of the atlas have been also observed associated with conditions such as Down syndrome [76, 77], Chiari malformation [78], thalassemia minor [79], Klippel-Feil syndrome [80–82]and other skeletal dysplasias like Goldenhar syndrome, Conradi syndrome, and atlas assimilation [74]. Focusing on the type A defect, most of the reported cases correspond to incidental findings during routine medical examination [37, 51, 83], with some exceptional cases of a type A associated with clinical manifestations (torticollis, facial asymmetry) [84]. With regard to anterior clefts of the atlas, in most cases a bipartite atlas or an isolated anterior cleft again constitutes an incidental finding during clinical examination subsequent to trauma to head and neck [56, 57, 61–72, 85, 86], and only in a few cases is an anterior cleft of the atlas associated with clinical symptoms, for instance due to hyperostosis caused by hypertrophy of the anterior arch [59]. Asrecently summarized [87], the review of the literature indicates that most of the bifid dorsal and ventral atlantal arches could be considered incidental findings, and thus likely no clinical symptoms would have been associated with the congenital clefts of SD-1643 and SD-1094.

Interestingly, defects of the arch of the atlas have been observed in two family groups, a mother (type B) and her sibling (type E) [47], and a mother (type A) and her two siblings (type E plus anterior cleft of the atlas in both cases) [84], in the latter case a condition compatible with autosomal dominant inheritance. At El Sidrón, a dental anomaly (retained left mandibular deciduous canine) with a potential heritable basis has been previously described for two individuals who presented the same mitochondrial haplotype [24], and in this regard statistically significant higher frequencies of type A defects of the atlas have been associated consistently with cleft palate [88, 89]and palatally displaced canines [90]. Other dental anomalies (i.e. uneruption and transposition of teeth) have been incidentally associated with both anterior and posterior clefts of the atlas [91, 92]. The association between clefts of the atlas with dental and other cervical anomalies [60, 62, 64, 80], most likely indicates a disturbance common to the close periods of embryogenesis of these structures. In this regard, a thorough and explanatory embryology-based classification of bony malformations of the craniovertebral junction has been elaborated by Pang and Thompson [87], who indicate that malformations of the arch of the atlas emerge from disturbances of the lateral zone of the upper cervical sclerotome (posterior arch) and the hypochordal bow (anterior arch). Further research in the embryological origin and schedule of cervical and craniofacial structures would shed light on these associations.

### Potential implications for Neandertal demography

We present here the first report of congenital clefts of the arch of the atlas in Neandertals. No other cases of such defects have been observed in studies of Neandertal cervical vertebrae which include those cases with best preservation of the atlas such as La Ferrassie 1 [93], Krapina 100–101[94, 95], and Regourdou 1 [96], or in studies of other well preserved hominin atlases [97]. Based solely on the findings for the atlas presented here and on the previously reported cases of dental anomalies [24], the number of individuals from El Sidrón presenting congenital conditions would range from a minimum of two (Adolescent 3/SD-1643, an association supported by compatible dental and bone age; Adult 2/SD-1094), to a maximum of four (each atlas and tooth representing a different individual). The observation that at least two out of three observable atlases present low frequency congenital anomalies supports previous genetic [11] and dental [24] evidence from El Sidrón indicating that these Neandertals constituted a group with close genetic relations. Familial relationships have been previously proposed for human skeletal remains in archaeological [21] and forensic [98] contexts, based on the common presence of anatomical variants of low population frequency. Although most of the variants used in the detection of kin groups are dental traits, cranial [99] and postcranial variations including traits of the first cervical vertebra [100, 101] have been also reported to be associated to familial relations. But beyond the usefulness of these traits for recognition of kin groups, it has been suggested in conservation biology that some anomalies without direct effect on fitness (e.g. minor tooth anomalies and thoracolumbar and sacrococygeal transitional vertebrae), could be used as indication of inbreeding [102]. In this regard, González-Reimers et al. [103] observed a bipartite atlas, an atlas with a type B defect, and two cervical blocks in a prehispanic ossuary of the island of El Hierro (Canary Islands), pointing to possible familial relations or to an unusually high prevalence of these conditions in this islander population. Similar observations have been reported by Merbs[104] in his study of vertebral developmental errors in Canadian Inuit skeletons, where a higher frequency and intensity of several spine defects were observed in the smaller and more genetically isolated of the two compared populations. While for North American islander prehistoric populations, it has been suggested that the high prevalence of maxillary canine-premolar transposition could be a signal of endogamy[22]. A more direct approach to inbreeding has been presented by Palma and Carini[105] and Alt et al[23]. The former authors observed a high frequency of cervical ribs at the 7^th^cervical vertebra in an isolated population from Sicily, while the latter observed a high frequency (35.7%) of congenitally missing maxillary lateral incisors in a 9000-year-old late Pre-Pottery Neolithic community in Southern Jordan. After a thorough review of the literature, these authors suggest that this could only be explained by close familial relationships, in this case due to socio-cultural choice of endogamy.

In general, skeletal findings suggesting inbreeding would fit the demographic scenario for early humans, with small size groups, population dispersal and potentially significant levels of intragroup and intrafamily mating resulting in high levels of consanguinity through time [106]. In this respect, and with regard to a very rare anomaly observed in the parietal bones of Xujiayao 11, an early Late Pleistocene fossil from China, Wu et al. [107] review the unusually high incidence of rare conditions among Pleistocene humans. These authors suggest the possibility that the high frequency of these conditions could reflect small and highly inbred populations during the Pleistocene, and the congenital clefts of the atlas from the Neandertals of El Sidrón would fit this broader scenario for human population dynamics during the Pleistocene. Specifically for Neandertals, as recently summarized [20], a similar general picture emerges from recent advances in paleogenetics, which posits a long-term small and decreasing population size sometime after 0.5–1.0 million years ago, with reduced and isolated groups. The consequence would be increased inbreeding at times. For instance, genetic analysis from two Neandertal individuals, from El Sidrón and Vindija respectively, indicate that mating among related individuals may have been more common in Neandertals than in present-day humans [108]. This observation would join with genetic evidence from a Neandertal woman from Siberia indicating a close familial relation between her parents (e.g. half-siblings with a common mother), further analysis pointing to frequent mating between closely related individuals in Neandertals from this geographic area [3]. Thus, an additional potential interpretation of the findings of dental and skeletal congenital anomalies at El Sidrón could be a signal of inbreeding in Neandertals from this geographic area. Although this inference would fit the general scenario emerging from skeletal studies [107] of Pleistocene humans and Neandertal paleogenetics [20], it is clear that further skeletal findings are needed in order to support this interpretation. In this regard, supernumerary ribs associated to the first lumbar vertebra, a low frequency developmental defect in modern humans, have been documented for two Neandertal individuals, Shanidar 3 and Kebara 2 [109, 110].

It is important to note, however, that inbreeding is not the only possible explanation for a high incidence of congenital conditions, which could also be explained by adverse environmental conditions impacting early pregnancies. For instance, congenital defects such as neural tube defects and orofacial clefts have been associated with socioeconomic status and maternal dietary patterns in epidemiological studies[111–113], while supernumerary ribs constitute a common finding in standard developmental toxicology bioassays [114]. Following both possibilities, it has been suggested that a combination of harsh environmental conditions and inbreeding was the most likely explanation for the high incidence in Late Pleistocene mammoths of cervical ribs[115], a congenital condition that in humans has been associated with multiple and major congenital abnormalities [116]. Studies of enamel hypoplasiaand dental fluctuating asymmetry indicate that Neandertal populations possibly suffered similar [117], or greater [118, 119] developmental stress than comparative prehistoric modern human samples.Specific evidence from El Sidrón indicates that all the dental individuals showed enamel hypoplasia, with well-marked defects on the incisors (59%), canines (50%), premolars (58%), and molars (32%) [7]. These data only partially support harsh environmental conditions as an explanatory factor for the presence of congenital conditions since they refer specifically to the period of crown formation, and not to the later period of pregnancy.

## Conclusions

We present the first two cases in Neandertals of a congenital posterior and anterior cleft of the arch of the atlas respectively. This observation, together with the previously reported presence of dental anomalies for two individuals from this site, could be interpreted as further evidence of the presence of a group with close genetic relations at El Sidrón, as a possible signal of inbreeding in this Neandertal group, and as an indication of harsh environmental conditions. Previous findings of high incidence of rare conditions among Pleistocene humans, and the general demographic scenario for Neandertals that emerges from paleogenetics would be compatible with inbreeding as an explanation of the presence of these low frequency clefts of the atlas in two of three observable atlases from El Sidrón. Further support or rejection ofthis and the aforementioned interpretations will come from new genetic and skeletal evidence from Neandertal remains.

## References

[1] Trinkaus E. Late Pleistocene adult mortality patterns and modern human establishment. Proceedings of the National Academy of Sciences of the United States of America. 2011;108(4):1267–71. 10.1073/pnas.1018700108 21220336 PMC3029716

[2] Fabre V, Condemi S, Degioanni A. Genetic evidence of geographical groups among Neanderthals. Plos One. 2009;4(4). 10.1371/journal.pone.0005151 PMC2664900 19367332

[3] Prufer K, Racimo F, Patterson N, Jay F, Sankararaman S, Sawyer S, et al The complete genome sequence of a Neanderthal from the Altai Mountains. Nature. 2014;505(7481):43–9. 10.1038/nature12886 24352235 PMC4031459

[4] Bocquet-Appel JP, Degioanni A. Neanderthal demographic estimates. Current Anthropology. 2013;54:S202–S13. 10.1086/673725

[5] Holliday TW, Gautney JR, Friedl L. Right for the wrong reasons reflections on modern human origins in the post-Neanderthal genome era. Current Anthropology. 2014;55(6):696–724. 10.1086/679068

[6] de la Rasilla M RA, Cañaveras JC, Lalueza-Fox C. La Cueva de El Sidrón (Borines, Piloñas, Asturias). Una investigación interdisciplinar de un grupo neandertal Oviedo: Gobierno del Prinicpado de Asturias; 2011.

[7] Rosas A, Martinez-Maza C, Bastir M, Garcia-Tabernero A, Lalueza-Fox C, Huguet R, et al Paleobiology and comparative morphology of a late Neandertal sample from El Sidrón, Asturias, Spain. Proc Natl Acad Sci U S A. 2006;103(51):19266–71. 10.1073/pnas.0609662104 17164326 PMC1748215

[8] Wood RE, Higham TFG, De Torres T, Tisnerat-Laborde N, Valladas H, Ortiz JE, et al A new date for the neanderthals from El Sidrón cave (Asturias, northern Spain). Archaeometry. 2013;55(1):148–58. 10.1111/j.1475-4754.2012.00671.x

[9] Rosas A, Estalrrich A, Garcia-Tabernero A, Bastir M, Garcia-Vargas S, Sanchez-Meseguer A, et al The Neandertals from El Sidrón (Asturias, Spain). Updating of a new sample. Anthropologie. 2012;116(1):57–76. 10.1016/j.anthro.2011.12.003

[10] Rosas A, Estalrrich A, Garcia-Vargas S, Garcia-Tabernero A, Huguet R, Lalueza-Fox C, et al Identification of Neandertal individuals in fragmentary fossil assemblages by means of tooth associations: the case of El Sidrón (Asturias, Spain). Comptes Rendus Palevol. 2013;12(5):279–91. 10.1016/j.crpv.2013.06.003

[11] Lalueza-Fox C, Rosas A, Estalrrich A, Gigli E, Campos PF, Garcia-Tabernero A, et al Genetic evidence for patrilocal mating behavior among Neandertal groups. Proceedings of the National Academy of Sciences of the United States of America. 2011;108(1):250–3. 10.1073/pnas.1011553108 21173265 PMC3017130

[12] Lalueza-Fox C, Gigli E, Sanchez-Quinto E, de la Rasilla M, Fortea J, Rosas A. Issues from Neandertal genomics: diversity, adaptation and hybridisation revised from the El Sidrón case study. Quaternary International. 2012;247:10–4. 10.1016/j.quaint.2010.10.012

[13] Hardy K, Buckley S, Collins MJ, Estalrrich A, Brothwell D, Copeland L, et al Neanderthal medics? Evidence for food, cooking, and medicinal plants entrapped in dental calculus. Naturwissenschaften. 2012;99(8):617–26. 10.1007/s00114-012-0942-0 22806252

[14] Estalrrich A, Rosas A. Division of labor by sex and age in Neandertals: an approach throu the study of activity-related dental wear. Journal of Human Evolution. 2015 80: 51–63. 10.1016/j.jhevol.2014.07.007 25681013

[15] Estalrrich A, Rosas A. Handedness in Neandertals from the El Sidrón (Asturias, Spain): evidence from instrumental striations with ontogenetic inferences. Plos One. 2013;8(5). 10.1371/journal.pone.0062797 PMC3646041 23671635

[16] Bastir M, Rosas A, Tabernero AG, Pena-Melian A, Estalrrich A, de la Rasilla M, et al Comparative morphology and morphometric assessment of the Neandertal occipital remains from the El Sidrón site (Asturias, Spain: years 2000–2008). Journal of Human Evolution. 2010;58(1):68–78. 10.1016/j.jhevol.2009.08.006 19836056

[17] Pena-Melian A, Rosas A, Garcia-Tabernero A, Bastir M, De La Rasilla M. Paleoneurology of Two new Neandertal occipitals from El Sidrón (Asturias, Spain) in the context of homo endocranial evolution. Anatomical Record-Advances in Integrative Anatomy and Evolutionary Biology. 2011;294(8):1370–81. 10.1002/ar.21427 21714107

[18] Bastir M, Garcia-Martinez D, Estalrrich A, Garcia-Tabernero A, Huguet R, Rios L, et al The relevance of the first ribs of the El Sidrón site (Asturias, Spain) for the understanding of the Neandertal thorax. Journal of Human Evolution. 2015;80:64–73. 10.1016/j.jhevol.2014.10.008 25563407

[19] Rosas A, Perez-Criado L, Bastir M, Estalrrich A, Huguet R, Garcia-Tabernero A, et al A geometric morphometrics comparative analysis of Neandertal humeri (epiphyses-fused) from the El Sidrón cave site (Asturias, Spain). J Hum Evol. 2015;82:51–66. 10.1016/j.jhevol.2015.02.018 25819346

[20] Sanchez-Quinto F, Lalueza-Fox C. Almost 20 years of Neanderthal palaeogenetics: adaptation, admixture, diversity, demography and extinction. Philosophical Transactions of the Royal Society B-Biological Sciences. 2015;370(1660). 10.1098/rstb.2013.0374 PMC4275882 25487326

[21] Alt KW, Pichler S, Vach W, Klima B, Vlcek E, Sedlmeier J. Twenty-five thousand-year-old triple burial from Dolni Vestonice: an ice-age family? American Journal of Physical Anthropology. 1997;102(1):123–31. 10.1002/(sici)1096-8644(199701)102:1<123::aid-ajpa10>3.0.co;2-2 9034043

[22] Sholts SB, Clement AF, Warmlander S. Brief communication: additional cases of maxillary canine-first premolar transposition in several prehistoric skeletal assemblages from the Santa Barbara Channel Islands of California. American Journal of Physical Anthropology. 2010;143(1):155–60. 10.1002/ajpa.21343 20564519

[23] Alt KW, Benz M, Muller W, Berner ME, Schultz M, Schmidt-Schultz TH, et al Earliest evidence for social endogamy in the 9,000-year-old-population of Basta, Jordan. Plos One. 2013;8(6). 10.1371/journal.pone.0065649 PMC3679157 23776517

[24] Dean MC, Rosas A, Estalrrich A, Garcia-Tabernero A, Huguet R, Lalueza-Fox C, et al Longstanding dental pathology in Neandertals from El Sidrón (Asturias, Spain) with a probable familial basis. Journal of Human Evolution. 2013;64(6):678–86. 10.1016/j.jhevol.2013.03.004 23615378

[25] AlQahtani SJ, Hector MP, Liversidge HM. Brief communication: The London Atlas of human tooth development and eruption. American Journal of Physical Anthropology. 2010;142(3):481–90. 10.1002/ajpa.21258 20310064

[26] Cardoso HFV, Garcia S. The not-so-dark ages: ecology for human growth in medieval and early twentieth century Portugal as inferred from skeletal growth profiles. American Journal of Physical Anthropology. 2009;138(2):136–47. 10.1002/ajpa.20910 18785651

[27] Wasterlain SN NM, Ferreira MT. Dental modifications in a skeletal sample of enslaved Africans found at Lagos (Portugal). International Journal of Osteoarchaeology. 2015 10.1002/oa.2453

[28] Ríos L, Perez M. Trauma perimortem en la maqbara medieval de Baza, Granada In: Roca de Togores C, Rodés F, editors. Jornadas de Antropología Física y Forense. Alicante, Spain: Instituto Alicantino de Cultura Juan Gil-Albert; 2007 p. 12–18.

[29] Ogden JA. Radiology of postnatal skeletal development.11. The 1st cervical vertebra. Skeletal Radiology. 1984;12(1):12–20. 6474212 10.1007/BF00373169

[30] Junewick JJ, Chin MS, Meesa IR, Ghori S, Boynton SJ, Luttenton CR. Ossification patterns of the atlas vertebra. American Journal of Roentgenology. 2011;197(5):1229–34. 10.2214/ajr.10.5403 22021519

[31] Piatt JH, Grissom LE. Developmental anatomy of the atlas and axis in childhood by computed tomography. Journal of Neurosurgery-Pediatrics. 2011;8(3):235–43. 10.3171/2011.6.peds11187 21882912

[32] Karwacki GM, Schneider JF. Normal ossification patterns of atlas and axis: a CT study. American Journal of Neuroradiology. 2012;33(10):1882–7. 10.3174/ajnr.A3105 22576894 PMC7964599

[33] Rao RD, Tang SJ, Lim C, Yoganandan N. Developmental morphology and ossification patterns of the C1 vertebra. Journal of Bone and Joint Surgery-American. 2013;95A(17):1605–. 10.2106/JBJS.L.01035 24005208

[34] Calvy TM, Segall HD, Gilles FH, Bird CR, Zee CS, Ahmadi J, et al CT anatomy of the craniovertebral junction in infants and children. American Journal of Neuroradiology. 1987;8(3):489–94. 3111211 PMC8331887

[35] Scheuer L BS. Developmental Juvenil Osteology. London: Academic Press; 2000.

[36] Cardoso HFV, Rios L. Age estimation from stages of epiphyseal union in the presacral vertebrae. American Journal of Physical Anthropology. 2011;144(2):238–47. 10.1002/ajpa.21394 20872802

[37] Kwon JK, Kim MS, Lee GJ. The incidence and clinical implications of congenital defects of atlantal arch. Journal of Korean Neurosurgical Society. 2009;46(6):522–7. 10.3340/jkns.2009.46.6.522 20062566 PMC2803266

[38] Geipel P. Studies on the fissure formation of the atlas and epistropheus. IV. Zentralblatt fur allgemeine Pathologie u pathologische Anatomie. 1955;94(1–2):19–84. 13282406

[39] Jefferson G. Fracture of the atlas vertebra—Report of four cases, and a review of those previously recorded. British Journal of Surgery. 1920;7(27):407–22.

[40] Gehweiler JA, Duff DE, Martinez S, Miller MD, Clark WM. Fractures of atlas vertebra. Skeletal Radiology. 1976;1(2):97–102.

[41] Segal LS, Grimm JO, Stauffer ES. Non-union of fractures of the atlas. Journal of Bone and Joint Surgery-American. 1987;69A(9):1423–34. 3440801

[42] Landells CD, Vanpeteghem PK. Fractures of the atlas—classification, treatment and morbidity. Spine. 1988;13(5):450–2. 10.1097/00007632-198805000-00002 3187689

[43] Levine AM, Edwards CC. Fractures of the Atlas. Journal of Bone and Joint Surgery-American. 1991;73A(5):680–91. 2045393

[44] Lee C, Woodring JH.Unstable jefferson variant atlas fractures—an unrecognized cervical injury. American Journal of Neuroradiology. 1991;12(6):1105–10. 1763734 PMC8331492

[45] Hu Y, Xu RM, Albert TJ, Vaccoro AR, Zhao HY, Ma WH, et al Function-preserving reduction and fixation of unstable Jefferson fractures using a C1 posterior limited construct. Journal of Spinal Disorders & Techniques. 2014;27(6):E219–E25. 24463337 10.1097/BSD.0b013e31829a36c5

[46] Sanchis-Gimeno JA, Blanco-Perez E, Aparicio L, Martinez-Soriano F, Martinez-Sanjuan V. Difficulties in distinguishing between an atlas fracture and a congenital posterior atlas arch defect in postmortem analysis. Forensic Science International. 2014;242:E1–E5. 10.1016/j.forsciint.2014.06.016 25037687

[47] Currarino G, Rollins N, Diehl JT. Congenital-defects of the posterior arch of the atlas—a report of 7 cases including an affected mother and son. American Journal of Neuroradiology. 1994;15(2):249–54. 8192068 PMC8334620

[48] Sanchis-Gimeno JA, Aparicio L. Posterior arch defect in a dry atlas. European Spine Journal. 2011;20(9):1574–5. 10.1007/s00586-011-1715-8 21327812 PMC3175901

[49] Jin M, Asadoorian M, Hiller LP, Hughes TH. Hypertrophy of the anterior arch of the atlas associated with congenital nonunion of the posterior arch: a retrospective case-control study. Spine J. 2014;14(7):1155–8. 10.1016/j.spinee.2013.07.482 24200414

[50] Guenkel S, Schlaepfer S, Gordic S, Wanner GA, Simmen HP, Werner CM. Incidence and variants of posterior arch defects of the atlas vertebra. Radiol Res Pract. 2013;2013:957280 10.1155/2013/957280 24109510 PMC3784273

[51] Senoglu M, Safavi-Abbasi S, Theodore N, Bambakidis NC, Crawford NR, Sonntag VKH. The frequency and clinical significance of congenital defects of the posterior and anterior arch of the atlas. Journal of Neurosurgery-Spine. 2007;7(4):399–402. 10.3171/spi-07/10/399 17933313

[52] Le Minor JM, Koritke JG. [Associations among non-metric features of the atlas in the human species]. Arch Anat Histol Embryol. 1991;74:11–26. 1366344

[53] Farman AG, Nortje CJ, Joubert JJ. Radiographic profile of the first cervical vertebra. J Anat. 1979;128(Pt 3):595–600. 468710 PMC1232910

[54] Desgrez H, Gentaz R, Chevrel JP. [Congenital abnormalities of the arcs of the atlas]. J Radiol Electrol Med Nucl. 1965;46(12):819–26. 5855309

[55] AF LD. Traité des variations de la colnne vertébrale de l'homme ed de leur signification au point de vue de l'anthropologie zoologique Paris: Vigot frères; 1912.

[56] Childers JC, Wilson FC. Bipartite atlas—Review of literature and report of a case. Journal of Bone and Joint Surgery-American. 1971;A 53(3):578–&. 4996286

[57] Lipson SJ, Mazur J. Anteroposterior spondyloschisis of atlas revealed bycomputerized tomography scanning—Case-report. Journal of Bone and Joint Surgery-American. 1978;60(8):1104–5. 721859

[58] Haakonsen M, Gudmundsen TE, Histol O. Midline anterior and posterior atlas clefts may simulate a jefferson fracture—a report of 2 cases. Acta Orthopaedica Scandinavica. 1995;66(4):369–71. 7676829 10.3109/17453679508995564

[59] Walker J, Beggs I. Bipartite atlas and hypertrophy of its anterior arch—a Case-report. Acta Radiologica. 1995;36(2):152–3. 7710794

[60] Atasoy C, Fitoz S, Karan B, Erden I, Akyar S. A rare cause of cervical spinal stenosis: posterior arch hypoplasia in a bipartite atlas. Neuroradiology. 2002;44(3):253–5. 10.1007/s00234-001-0740-4 11942383

[61] Prempeh RC, Gibson JC, Bhattacharya JJ. Mid-line clefts of the atlas: a diagnostic dilemma. Spinal Cord. 2002;40(2):92–3. 10.1038/sj.sc.3101230 11926422

[62] Garg A, Gaikwad SB, Gupta V, Mishra NK, Kale SS, Singh J. Bipartite atlas with os odontoideum—Case report. Spine. 2004;29(2):E35–E8. 10.1097/01.brs.0000106487.89648.88 14722424

[63] Osher SJ, Nasser NA. Coincidental finding of a bipartite atlas during assessment of facial trauma. British Journal of Oral & Maxillofacial Surgery. 2004;42(3):270–1. 10.1016/j.bjoms.2004.01.006 15121280

[64] Osti M, Philipp H, Meusburger B, Benedetto KP. Os odontoideum with bipartite atlas and segmental instability: a case report. Eur Spine J. 2006;15 Suppl 5:564–7. 10.1007/s00586-005-0017-4 16311753 PMC1602181

[65] Muthu SK, Cox S, Gunawardena WJ, Balakrishnan G. Anteroposterior spondyloschisis of the atlas. Two case reports and literature review. Emerg Radiol. 2007;13(6):337–40. 10.1007/s10140-006-0565-x 17252248

[66] Jans C, Mahieu G, Van Riet R. Bipartite atlas mimicking traumatic atlantoaxial instability following a rugby tackle. BMJ Case Rep. 2009;2009. 10.1136/bcr.04.2009.1824 PMC3027740 21857880

[67] Hu Y, Ma WH, Xu RM. Transoral osteosynthesis C1 as a function-preserving option in the treatment of bipartite atlas deformity. A case report. Spine. 2009;34(11):E418–E21. 10.1097/BRS.0b013e3181a0ff2f. 19444056

[68] Park JS, Eun JP, Lee HO. Anteroposterior spondyloschisis of atlas with bilateral cleft defect of posterior arch.A case report. Spine. 2011;36(2):E144–E7. 10.1097/BRS.0b013e3181efa320 21228693

[69] Petre BM, Karp JE, Riley LH. Athletic cervical spine injury in the setting of fusion failure of the anterior and posterior atlas. Orthopedics. 2012;35(9):E1449–E52. 10.3928/01477447-20120822-39 22955419

[70] Petraglia AL, Childs SM, Walker CT, Hogg J, Bailes JE, Lively MW. Bipartite atlas in a collegiate football player—Not necessarily a contraindication for return-to-play: A case report and review of the literature. Surg Neurol Int. 2012;3:126 10.4103/2152-7806.102351 23227431 PMC3513844

[71] Ganau M, Spinelli R, Tacconi L. Complex developmental abnormality of the atlas mimicking a Jefferson fracture: Diagnostic tips and tricks. J Emerg Trauma Shock. 2013;6(1):47–9. 10.4103/0974-2700.106325. 23493176 PMC3589859

[72] Hummel E, de Groot JC. Three cases of bipartition of the atlas. Spine Journal. 2013;13(5):E1–E5. 10.1016/j.spinee.2013.01.024 23415018

[73] Tyrrell AJ, Benedix DC. Two cases of atlar anomalies. International Journal of Osteoarchaeology. 2004;14(1):52–9. 10.1002/oa.712

[74] Menezes AH. Craniocervical developmental anatomy and its implications. Childs Nervous System. 2008;24(10):1109–22. 10.1007/s00381-008-0600-1 18401563

[75] Klimo P, Blumenthal DT, Couldwell WT. Congenital partial aplasia of the posterior arch of the atlas causing myelopathy: Case report and review of the literature. Spine. 2003;28(12):E224–E8. 10.1097/00007632-200306150-00023 12811285

[76] Martich V, Benami T, Yousefzadeh DK, Roizen NJ. Hypoplastic posterior arch of c-1 in children with down-syndrome—a double jeopardy. Radiology. 1992;183(1):125–8. 1532260 10.1148/radiology.183.1.1532260

[77] Pascual-Gallego M, Budke M, Villarejo F. Spinal stenosis at the level of atlas in a boy with Down syndrome. A case report and literature review. Neurocirugia. 2014;25(1):29–32. 10.1016/j.neucir.2012.10.002 23218994

[78] Wang SL, Wang C, Yan M, Zhou HT, Jiang L. Syringomyelia with irreducible atlantoaxial dislocation, basilar invagination and Chiari I malformation. European Spine Journal. 2010;19(3):361–6. 10.1007/s00586-009-1208-1 19941013 PMC2899758

[79] Schrodel MH, Braun V, Stolpe E, Hertlein H. Coincidental deficiency of the posterior arch of the atlas and thalassaemia minor: possible pitfalls in a trauma victim. Emergency Medicine Journal. 2005;22(7):526–8. 10.1136/emj.2003.006759 15983100 PMC1726848

[80] Wolf RFE, Klein JP. Complete bipartition of the atlas in the Klippel-Feil syndrome—A radiologically illustrated case report. Surgical and Radiologic Anatomy. 1997;19(5):339–40. 10.1007/bf01637605 9413084

[81] Martirosyan NL, Cavalcanti DD, Kalani MYS, Maughan PH, Theodore N, Pritchard PR. Aplasia of the anterior arch of atlas associated with multiple congenital disorders: case report. Neurosurgery. 2011;69(6):E1317–E20. 10.1227/NEU.0b013e31822a9ab1 21712741

[82] Dilettoso S, Uccello M, Dilettoso A, Gelardi S, Dilettoso B. Duplicated odontoid process and atlas clefts associated to Klippel-Feil syndrome. Spine Journal. 2012;12(5):449–50. 10.1016/j.spinee.2012.03.031 22513073

[83] Blanco-Perez E, Sanchez-Jurado R, Mata-Escolano F, Sanchis-Gimeno JA. Congenital failure of midline fusion of the posterior atlas arch with an associated unilateral cleft. Spine Journal. 2015;15(1):198–9. 10.1016/j.spinee.2014.08.450 25200329

[84] Al Kaissi A, Ben Chehida F, Gharbi H, Ben Ghachem M, Grill F, Varga F, et al Persistent torticollis, facial asymmetry, grooved tongue, and dolicho-odontoid process in connection with atlas malformation complex in three family subjects. European Spine Journal. 2007;16:S265–S70. 10.1007/s00586-006-0297-3 PMC2148083 17245565

[85] Chalmers AG, Gallegos NC. Spondyloschisis of the anterior arch of the atlas. British Journal of Radiology. 1985;58(692):761–3. 3842280 10.1259/0007-1285-58-692-761

[86] Sasaka KK, Decker GT, El-Khoury GY. Horizontal fracture of the anterior arch of the atlas associated with a congenital cleft of the anterior arch. Emerg Radiol. 2006;12(3):130–2. 10.1007/s10140-005-0455-7 16429318

[87] Pang D, Thompson DNP. Embryology, classification, and sugical management of bony malformations of the craniovertebral junction In: Di Rocco C, Akalan N, editors. Pediatric craniovertebral junction diseases. Surgical management of craniovertebral junction diseases in children Switzerland: Springer; 2014 pp. 19–110.

[88] Sandham A. Cervical vertebral anomalies in cleft-lip and palate. Cleft Palate Journal. 1986;23(3):206–14. 3524906

[89] Ugar DA, Semb G. The prevalence of anomalies of the upper cervical vertebrae in subjects with cleft lip, cleft palate, or both. Cleft Palate-Craniofacial Journal. 2001;38(5):498–503. 10.1597/1545-1569(2001)038<0498:tpoaot>2.0.co;2 11522172

[90] Leonardi R, Barbato E, Vichi M, Caltabiano M. Skeletal anomalies and normal variants in patients with palatally displaced canines. Angle Orthodontist. 2009;79(4):727–32. 10.2319/082408-448.1 19537879

[91] Popat H, Drage N, Durning P. Mid-line clefts of the cervical vertebrae—An incidental finding arising from cone beam computed tomography of the dental patient. British Dental Journal. 2008;204(6):303–6. 10.1038/bdj.2008.199 18356876

[92] Rogers SA, Drage N, Durning P. Incidental findings arising with cone beam computed tomography imaging of the orthodontic patient. Angle Orthodontist. 2011;81(2):350–5. 10.2319/032210-165.1 21208090 PMC8925247

[93] Gomez-Olivencia A. The presacral spine of the La Ferrasie 1 Neandertal: a revised inventory. Bulletins et mémoires de la Societé d'anthropologie de Paris. 2013;25(1–2):19–38.

[94] Radovcic J SF, Trinkaus E, Wolpoff M. The Krapina hominids: an illustrated catalog of the skeletal collection Zagreb: Mladost and the Croatian Natural History Museum; 1988.

[95] Gomez-Olivencia A, Been E, Arsuaga JL, Stock JT. The Neandertal vertebral column 1: The cervical spine. Journal of Human Evolution. 2013;64(6):608–30. 10.1016/j.jhevol.2013.02.008 23541382

[96] Gomez-Olivencia A, Couture-Veschambre C, Madelaine S, Maureille B. The vertebral column of the Regourdou 1 Neandertal. Journal of Human Evolution. 2013;64(6):582–607. 10.1016/j.jhevol.2013.02.006 23566460

[97] Gomez-Olivencia A, Carretero JM, Arsuaga JL, Rodriguez-Garcia L, Garcia-Gonzalez R, Martinez I. Metric and morphological study of the upper cervical spine from the Sima de los Huesos site (Sierra de Atapuerca, Burgos, Spain). Journal of Human Evolution. 2007;53(1):6–25. 10.1016/j.jhevol.2006.12.006 17467038

[98] Rios L, Ovejero JIC, Prieto JP. Identification process in mass graves from the Spanish Civil War I. Forensic Science International. 2010;199(1–3):E27–E36. 10.1016/j.forsciint.2010.02.023 20399578

[99] Z Z. New method of idenifying family related skulls: forensic medicine, anthropology, epigenetics Springer; 2005.

[100] Selby S, Garn SM, Kanareff V. The incidence and familial nature of a bony bridge on the first cervical vertebra. Am J Phys Anthropol. 1955;13(1):129–41. 14361662 10.1002/ajpa.1330130110

[101] Saunders SR, Popovich F. A family study of two skeletal variants: atlas bridging and clinoid bridging. Am J Phys Anthropol. 1978;49(2):193–203. 10.1002/ajpa.1330490207 717554

[102] Raikkonen J, Vucetich JA, Vucetich LM, Peterson RO, Nelson MP. What the inbred Scandinavian wolf population tells us about the nature of conservation. Plos One. 2013;8(6). 10.1371/journal.pone.0067218 PMC3689695 23805301

[103] Gonzalez-Reimers E, Mas-Pascual A, Arnay-De-La-Rosa M, Velasco-Vazquez J, Jimenez-Gomez MC. Klippel-Feil syndrome in the prehispanic population of El Hierro (Canary Islands). Annals of the Rheumatic Diseases. 2001;60(2):174–. 10.1136/ard.60.2.173a PMC1753466 11203718

[104] Merbs CF. Sagittal clefting of the body and other vertebral developmental errors in Canadian Inuit skeletons. Am J Phys Anthropol. 2004;123(3):236–49. 10.1002/ajpa.10264 14968421

[105] Palma A, Carini F. [Variation of the transverse apophysis of the 7th cervical vertebra: anatomo-radiological study of an isolated population]. Arch Ital Anat Embriol. 1990;95(1):11–6. 2275597

[106] Bittles AH, Black ML. Consanguineous marriage and human evolution. In: Brenneis D, Ellison PT, editors. Annual Review of Anthropology, Volume 39. Annual Review of Anthropology. 392010. p. 193–207.

[107] Wu X-J, Xing S, Trinkaus E. An enlarged parietal foramen in the Late Archaic Xujiayao 11 neurocranium from Northern China, and rare anomaliesamong Pleistocene Homo. Plos One. 2013;8(3): e59587 10.1371/journal.pone.0059587 23527224 PMC3601107

[108] Castellano S, Parra G, Sanchez-Quinto FA, Racimo F, Kuhlwilm M, Kircher M, et al Patterns of coding variation in the complete exomes of three Neandertals. Proceedings of the National Academy of Sciences of the United States of America. 2014;111(18):6666–71. 10.1073/pnas.1405138111 24753607 PMC4020111

[109] Ogilvie MD, Hilton CE, Ogilvie CD. Lumbar anomalies in the Shanidar 3 Neandertal. Journal of Human Evolution. 1998;35(6):597–610. 10.1006/jhev.1998.0249 9929171

[110] Eaves-Johnson KL. Supernumerary lumbar rib in human prehistory. Faseb Journal. 2010;24.

[111] Carmichael SL, Yang W, Herring A, Abrams B, Shaw GM. Maternal food insecurity is associated with increased risk of certain birth defects. Journal of Nutrition. 2007;137(9):2087–92. 17709447 10.1093/jn/137.9.2087 PMC2063452

[112] Yang J, Carmichael SL, Canfield M, Song J, Shaw GM. Socioeconomic status in relation to selected birth defects in a large multicentered US case-control study. American Journal of Epidemiology. 2008;167(2):145–54. 10.1093/aje/kwm283 17947220

[113] Carmichael SL, Yang W, Feldkamp ML, Munger RG, Siega-Riz AM, Botto LD, et al Reduced risks of neural tube defects and orofacial clefts with higher diet quality. Archives of Pediatrics & Adolescent Medicine. 2012;166(2):121–6. 10.1001/archpediatrics.2011.185 21969361 PMC3973484

[114] Chernoff N, Rogers JM. Supernumerary ribs in developmental toxicity bioassays and in human populations: incidence and biological significance. J Toxicol Environ Health B Crit Rev. 2004;7(6):437–49. 10.1080/10937400490512447 15586878

[115] Reumer JW, Ten Broek CM, Galis F. Extraordinary incidence of cervical ribs indicates vulnerable condition in Late Pleistocene mammoths. PeerJ. 2014;2:e318 10.7717/peerj.318 24711969 PMC3970796

[116] Galis F, Van Dooren TJM, Feuth JD, Metz JAJ, Witkam A, Ruinard S, et al Extreme selection in humans against homeotic transformations of cervical vertebrae. Evolution. 2006;60(12):2643–54. 17263123

[117] Guatelli-Steinberg D, Larsen CS, Hutchinson DL. Prevalence and the duration of linear enamel hypoplasia: a comparative study of Neandertals and Inuit foragers. J Hum Evol. 2004;47(1–2):65–84. 10.1016/j.jhevol.2004.05.004 15288524

[118] Ogilvie MD, Curran BK, Trinkaus E. Incidence and patterning of dental enamel hypoplasia among the Neanderthals. American Journal of Physical Anthropology. 1989;79(1):25–41. 10.1002/ajpa.1330790104 2665513

[119] Barrett CK, Guatelli-Steinberg D, Sciulli PW. Revisiting dental fluctuating asymmetry in neandertals and modern humans. American Journal of Physical Anthropology. 2012;149(2):193–204. 10.1002/ajpa.22107 22791408

